# Contribution of Phase Resetting to Adaptive Rhythm Control in Human Walking Based on the Phase Response Curves of a Neuromusculoskeletal Model

**DOI:** 10.3389/fnins.2020.00017

**Published:** 2020-02-05

**Authors:** Daiki Tamura, Shinya Aoi, Tetsuro Funato, Soichiro Fujiki, Kei Senda, Kazuo Tsuchiya

**Affiliations:** ^1^Department of Aeronautics and Astronautics, Graduate School of Engineering, Kyoto University, Kyoto, Japan; ^2^Department of Mechanical Engineering and Intelligent Systems, Graduate School of Informatics and Engineering, The University of Electro-Communications, Tokyo, Japan; ^3^Department of Physiology and Biological Information, School of Medicine, Dokkyo Medical University, Tochigi, Japan

**Keywords:** human walking, phase resetting, phase response curve, central pattern generator, muscle synergy, neuromusculoskeletal model

## Abstract

Humans walk adaptively in varying environments by manipulating their complicated and redundant musculoskeletal system. Although the central pattern generators in the spinal cord are largely responsible for adaptive walking through sensory-motor coordination, it remains unclear what neural mechanisms determine walking adaptability. It has been reported that locomotor rhythm and phase are regulated by the production of phase shift and rhythm resetting (phase resetting) for periodic motor commands in response to sensory feedback and perturbation. While the phase resetting has been suggested to make a large contribution to adaptive walking, it has only been investigated based on fictive locomotion in decerebrate cats, and thus it remains unclear if human motor control has such a rhythm regulation mechanism during walking. In our previous work, we incorporated a phase resetting mechanism into a motor control model and demonstrated that it improves the stability and robustness of walking through forward dynamic simulations of a human musculoskeletal model. However, this did not necessarily verify that phase resetting plays a role in human motor control. In our other previous work, we used kinematic measurements of human walking to identify the phase response curve (PRC), which explains phase-dependent responses of a limit cycle oscillator to a perturbation. This revealed how human walking rhythm is regulated by perturbations. In this study, we integrated these two approaches using a physical model and identification of the PRC to examine the hypothesis that phase resetting plays a role in the control of walking rhythm in humans. More specifically, we calculated the PRC using our neuromusculoskeletal model in the same way as our previous human experiment. In particular, we compared the PRCs calculated from two different models with and without phase resetting while referring to the PRC for humans. As a result, although the PRC for the model without phase resetting did not show any characteristic shape, the PRC for the model with phase resetting showed a characteristic phase-dependent shape with trends similar to those of the PRC for humans. These results support our hypothesis and will improve our understanding of adaptive rhythm control in human walking.

## 1. Introduction

Humans walk adaptively in varying environments by the skillful control of their complicated and redundant musculoskeletal system. Although many studies have investigated the underlying mechanism for adaptive walking, it remains largely unclear what neural mechanisms determine the walking adaptability.

Because human walking is rhythmic, elucidating the rhythm control strategy is crucial. The central pattern generators (CPGs) in the spinal cord are largely responsible for adaptive rhythm control through sensory-motor coordination (Orlovsky et al., 1999). In particular, it has been reported that locomotor rhythm and phase are regulated by producing phase shift and rhythm resetting (phase resetting) for periodic motor commands in response to sensory feedback and perturbation (Duysens, 1977; Conway et al., 1987; Guertin et al., 1995; Schomburg et al., 1998; Lafreniere-Roula and McCrea, 2005; Rybak et al., 2006a; Frigon and Gossard, 2010). However, such phase resetting behavior has been investigated only with electromyographic and electroneurographic data measured during fictive locomotion in decerebrate cats, and thus it is unclear if human motor control has such a rhythm regulation mechanism during walking. From a modeling approach on the basis of the hypothesis that phase resetting works for the control of walking rhythm in humans, the phase resetting mechanism has been introduced in motor control models of human walking. Although the models demonstrated that it improves stability and robustness of walking through forward dynamic simulations of human musculoskeletal models (Yamasaki et al., 2003a,b; Nomura et al., 2009; Aoi et al., 2010; Aoi and Funato, 2016), they did not necessarily verify whether the hypothesis is true.

To investigate rhythm regulation mechanisms in biological and natural phenomena, researchers have applied the phase response curve (PRC) in the phase reduction theory, which explains how the phase of a limit cycle oscillator shifts by a perturbation at an arbitrary phase (Kuramoto, 1984; Winfree, 2001). In our previous work (Funato et al., 2016), we assumed human walking as a limit cycle oscillator and identified the PRC from kinematic measurements by changing the belt speed of a treadmill during human walking, which clarified how human walking rhythm is regulated by perturbations. In this study, to examine the hypothesis, we integrated two previous different approaches that used a physical model and identification of the PRC. More specifically, we performed forward dynamic simulations with our previous neuromusculoskeletal model (Aoi et al., 2010) to walk on a treadmill and disturbed the belt speed at arbitrary phases in the same way as our previous experiments with humans (Funato et al., 2016). In particular, we obtained the PRC for two different cases with and without phase resetting in our motor control model and compared the results with the measured PRC in humans. Based on these results, we discuss the contribution of phase resetting to adaptive rhythm control in human walking.

## 2. Methods

### 2.1. Model

In this study, we used the same neuromusculoskeletal model that we developed in our previous work (Aoi et al., 2010). We briefly explain the model below.

#### 2.1.1. Musculoskeletal Model

**Figure 1:**
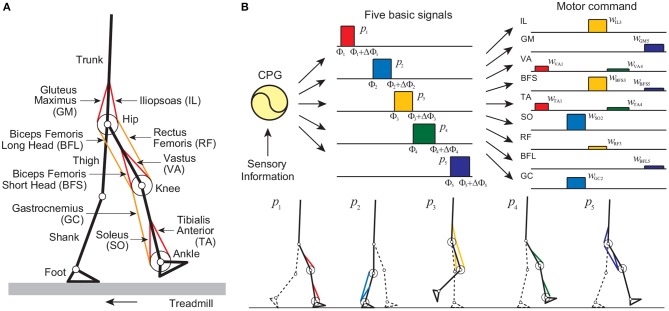
Neuromusculoskeletal model: **(A)** musculoskeletal model walking on a treadmill and **(B)** motor command composed of the linear combination of five rectangular pulses based on the muscle synergy hypothesis, and identification of the muscles activated by each pulse. Each sole has four contact points (two for the toe part and the others for the heel part) to receive reaction forces from the treadmill belt through linear spring and damper systems for each point.

Our musculoskeletal model is two-dimensional (Figure 1A), and the physical parameters were determined from data obtained from measurement of human walking (Davy and Audu, 1987; Winter, 2004). The skeletal part of our model has seven rigid links: trunk (head, arms, and torso) and thigh, shank, and foot of each leg, and has nine degrees of freedom: hip, knee, and ankle joint angles of each leg and horizontal and vertical translations and rotation of the trunk. Each joint has a linear viscous element, and the knee and ankle joints are subject to large linear elastic and damping torques when these joint angles exceed their limits. We used four contact points on each sole to receive reaction forces from the treadmill belt (toe, heel, and 4.0 cm inside from the toe and from the heel). The reaction force is modeled by a linear spring and damper system for each horizontal and vertical direction. Our model contains nine principal muscles to achieve the necessary motions in each leg. Six muscles produce uniarticular motion: hip flexion [iliopsoas (IL)], hip extension [gluteus maximus (GM)], knee extension [vastus (VA)], knee flexion [biceps femoris short head (BFS)], ankle flexion [tibialis anterior (TA)], and ankle extension [soleus (SO)]. Three muscles produce biarticular motion: hip flexion and knee extension [rectus femoris (RF)], hip extension and knee flexion [biceps femoris long head (BFL)], and knee flexion and ankle extension [gastrocnemius (GC)]. The muscle model consists of contractile and passive elements. The contractile part depends on force-length and force-velocity relationships and the muscle activation, which is determined through a low-pass filtering of the motor command *u*_*m*_ (*m* = IL, GM, VA, BFS, TA, SO, RF, BFL, and GC) from the motor control model. The equations of motion in this model were derived using Lagrangian mechanics and solved using the fourth-order Runge-Kutta method with time steps of 2 × 10^−7^ s for the forward dynamic simulation.

#### 2.1.2. Motor Control Model

Our motor control model consists of a hypothetical two-layered CPG model at the spinal cord level, which incorporates phase resetting, and a movement regulation model at the brainstem and cerebellum levels. The CPGs in the spinal cord have been suggested to consist of hierarchical networks that include rhythm generator (RG) and pattern formation (PF) networks (Burke et al., 2001; Lafreniere-Roula and McCrea, 2005; Rybak et al., 2006a,b). The RG network generates the basic rhythm and alters it by producing phase shifts and rhythm resetting in response to sensory feedback, while the PF network shapes the rhythm into spatiotemporal patterns of motoneuron activities. For the RG model, we used two simple oscillators whose phase is ϕ_*i*_ (0 ≤ ϕ_*i*_ < 2π, *i* = right, left) to produce the basic rhythm of the corresponding leg and incorporated phase resetting as explained below. For the PF model, we determined motor commands necessary to produce periodic leg movements in accordance with the oscillator phase based on the muscle synergy hypothesis, which suggests that the linear combination of five basic signals produces a large portion of the motor commands for human locomotion (Ivanenko et al., 2006). More specifically, we used five rectangular pulses *p*_*i*_(ϕ) (*i* = 1, …, 5) for each leg (Figure 1B), which are given by:

(1) $$\begin{array}{l} p_{i}\left(\phi \right)=\left\{ \begin{array}{cc} 1 & \Phi_{i}<\phi \leq \Phi_{i}+\Delta \Phi_{i}\\ 0 & \text{otherwise} \end{array}\right.i=1,\ldots ,5 \end{array}$$

where Φ_*i*_ and ΔΦ_*i*_ (*i* = 1, …, 5) are the onset phase and duration, respectively, of the rectangular pulses, and we omitted the suffix of ϕ. We determined the muscle synergy-based motor command $u_{m}^{\text{Syn}}$ by:

(2) $$\begin{array}{l} u_{m}^{\text{Syn}}=\overset{5}{\underset{i=1}{\sum }}w_{m,i}\Lambda_{i}p_{i}\left(\phi \right) \end{array}$$

where *w*_*m, i*_ (*i* = 1, …, 5) is the weighting coefficient of five rectangular pulses (*w*_*m, i*_ ≥ 0) and Λ_*i*_ (*i* = 1, …, 5) is the tuning parameter of the amplitude of the rectangular pulses for different belt speeds.

To emulate the phase shift and rhythm resetting behavior, we incorporated the phase resetting mechanism in the RG model. More specifically, we reset the oscillator phase to a nominal value based on foot contact information by using the following phase dynamics:

(3) $$\begin{array}{l} {\overset{∙}{\phi }}_{i}=\omega -K_{\phi }\text{ sin}\left(\Delta \phi_{i}-\pi \right)-\left(\phi_{i}-\phi^{\text{FC}}\right)\delta \left(t-t_{i}^{\text{FC}}-\tau^{\text{FC}}\right) \end{array}$$

where,

$$\begin{array}{l} \Delta \phi_{i}=\left\{ \begin{array}{cc} \phi_{\text{right}}-\phi_{\text{left}} & i=\text{right}\\ \phi_{\text{left}}-\phi_{\text{right}} & i=\text{left} \end{array}\right. \end{array}$$

ω is the basic frequency, *K*_ϕ_ is the gain parameter, $t_{i}^{\text{FC}}$ is the foot-contact time, τ^FC^ (= 50 ms) is the transmission delay in receiving the foot-contact information, ϕ^FC^ is the phase value to be reset at the foot contact, and δ(·) is the Dirac delta function. The second term on the right-hand side maintains interlimb coordination so that the legs move in antiphase. The third term of the right-hand side corresponds to phase resetting, which resets the oscillator phase ϕ_*i*_ to ϕ^FC^ to modulate the timing of the muscle synergy-based motor command based on the foot contact information. The second and third terms regulate the gait frequency and contribute to the generation of stable limit cycle for walking.

In addition to the CPG model at the spinal cord level, we used a movement regulation model at the brainstem and cerebellum levels based on somatosensory information, where only two crucial factors were incorporated for simplicity: maintenance of an upright posture and the desired forward speed. For the maintenance of an upright posture, a simple feedback control regulates the balance of the trunk pitch to prevent it from falling over using antagonistic uniarticular muscles in the hip of the standing leg.

(4) $$\begin{array}{l} p_{m}^{\text{Trunk}}=\left\{ \begin{array}{cc} -\kappa_{m}\left(\theta -\hat{\theta }\right)-\sigma_{m}\overset{∙}{\theta } & \text{in stance phase}\\ 0 & \text{otherwise} \end{array}\right. \end{array}$$

where θ is the trunk pitch angle, $\overset{∙}{\theta }$ is the angular rate, $\hat{\theta }$ is the reference angle, and κ_*m*_ and σ_*m*_ are the gain parameters (κ_*m*_ = σ_*m*_ = 0 when *m* ≠ IL or GM). For maintenance of the speed, a simple feedback control is used to increase the ankle push-off when the speed is lower than desired and suppress the pushing force in the opposite case by antagonistic uniarticular muscles in the ankle of the standing leg.

(5) $$\begin{array}{l} p_{m}^{\text{Speed}}=\left\{ \begin{array}{cc} -\lambda_{m}\left(v-\hat{v}\right) & \text{in stance phase}\\ 0 & \text{otherwise} \end{array}\right. \end{array}$$

where *v* is the forward speed, $\hat{v}$ is the target forward speed, and λ_*m*_ is the gain parameter (λ_*m*_ = 0 when *m* ≠ TA or SO). Because these regulations operate at the brainstem and cerebellar levels, the command signals are delayed and the motor command $u_{m}^{\text{Reg}}$ is given by:

(6) $$\begin{array}{l} u_{m}^{\text{Reg}}\left(t\right)=p_{m}^{\text{Trunk}}\left(t-\tau^{\text{Reg}}\right)+p_{m}^{\text{Speed}}\left(t-\tau^{\text{Reg}}\right) \end{array}$$

where τ^Reg^ (= 80 ms) is the delay in receiving transmissions of somatosensory information at the brainstem and cerebellar levels and sending the motor command to the spinal cord level.

The motor command *u*_*m*_ is given by the summation of the muscle synergy-based motor command $u_{m}^{\text{Syn}}$ and the motor command by the movement regulation $u_{m}^{\text{Reg}}$.

(7) $$\begin{array}{l} u_{m}=u_{m}^{\text{Syn}}+u_{m}^{\text{Reg}} \end{array}$$

#### 2.1.3. Model Parameters

While the model in our previous work (Aoi et al., 2010) walked over the ground, the model in this study walked on a treadmill, as explained below. Therefore, we slightly modified the values of the motor control parameters so that the model achieved steady walking on the treadmill whose belt speed was 1.3 m/s as follows: the onset phase and duration of rectangular pulses were Φ_1_ = 6.12 rad, Φ_2_ = 1.48 rad, Φ_3_ = 2.56 rad, Φ_4_ = 3.51 rad, Φ_5_ = 5.38 rad, ΔΦ_1_ = 0.70 rad, ΔΦ_2_ = 0.90 rad, ΔΦ_3_ = 0.90 rad, ΔΦ_4_ = 1.07 rad, and ΔΦ_5_ = 0.96 rad, where we set ϕ = 0 rad at foot contact; the amplitudes and weighting coefficients of the rectangular pulses were Λ_*i*_ = 1.0 (*i* = 1, …, 5), *w*_VA,1_ = 0.42, *w*_TA,1_ = 0.35, *w*_SO,2_ = 1.26, *w*_GC,2_ = 0.87, *w*_IL,3_ = 1.02, *w*_BFS,3_ = 1.09, *w*_RF,3_ = 0.10, *w*_VA,4_ = 0.17, *w*_TA,4_ = 0.21, *w*_GM,5_ = 0.61, *w*_BFS,5_ = 0.20, *w*_BFL,5_ = 0.20, and the other *w*_*m,i*_ = 0; the parameters for the oscillator phase dynamics were ω = 2π/1.0 rad/s, *K*_ϕ_ = 1.7, and ϕ^FC^ = 0.36 rad; and the parameters for the movement regulation were κ_IL_ = −1.0, κ_GM_ = 2.0, σ_IL_ = −0.20, σ_GM_ = 0.40, λ_TA_ = −0.20, λ_SO_ = 0.12, $\hat{\theta }=-0.012$ rad, and $\hat{v}=0.1$ m/s.

For different belt speeds, we changed Φ_2_, ω, Λ_*i*_ (*i* = 1, …, 5), and ϕ^FC^ in a similar way to Aoi et al. (2019) as follows: Φ_2_ = 1.46 rad, ω = 2π/0.9 rad/s, Λ_1_ = 1.04, Λ_2_ = 1.14, Λ_3_ = 1.10, Λ_4_ = 1.03, Λ_5_ = 1.18, and ϕ^FC^ = 0.48 rad when the belt speed was increased by 0.02 m/s, and Φ_2_ = 1.50 rad, ω = 2π/1.1 rad/s, Λ_1_ = 0.96, Λ_2_ = 0.90, Λ_3_ = 0.90, Λ_4_ = 0.98, Λ_5_ = 0.82, and ϕ^FC^ = 0.04 rad when the belt speed was decreased by 0.02 m/s.

### 2.2. Phase Response Curve

**Figure 2:**
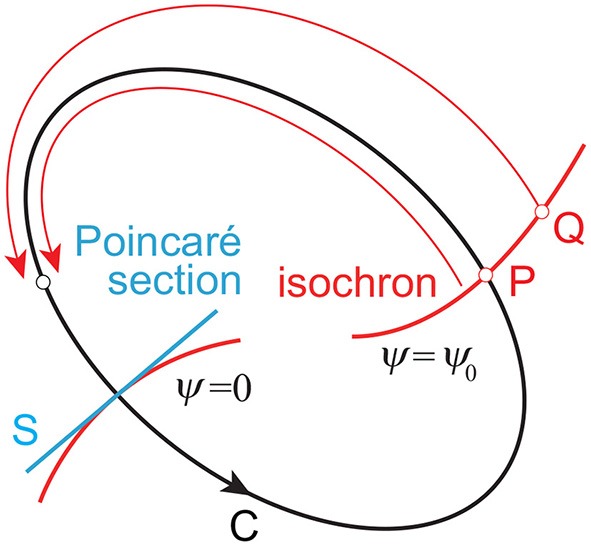
Limit cycle orbit C and isochron. Point P on C and point Q close to C converge to the same point on C for *t* → ∞ and are included in the same isochron. Poincaré section S, which determines the cycles, generally mismatches with any of the isochrons.

In the phase reduction theory (Kuramoto, 1984; Winfree, 2001), for a limit cycle oscillator whose period is τ and closed orbit is C on the phase space (Figure 2), we can define ψ on C, which follows the dynamics:

(8) $$\begin{array}{l} \overset{∙}{\psi }=\frac{2\pi }{\tau } \end{array}$$

To apply the phase dynamics to the neighborhood of the limit cycle, we assume that the point P on C and the point Q close to C have the same phase when they converge to the same point on C for *t* → ∞. The surface (curve) with the same phase (ψ = ψ_0_= const.) is called an isochron.

When a perturbation *I*(*t*) is added to the limit cycle oscillator, ψ follows the dynamics:

(9) $$\begin{array}{l} \overset{∙}{\psi }=\frac{2\pi }{\tau }+Z\left(\psi \right)I\left(t\right) \end{array}$$

where *Z*(ψ) is the PRC and explains the phase-dependent rhythm change due to the perturbation. We determine cycles using Poincaré section S, as shown in Figure 2. We assume that the trajectory converges to C before *I*(*t*) is added. We define *t* = 0 for the time at the last intersection of C with S before *I*(*t*) is added and ψ(0) = 0. We also define *t* = *t*_*n*_ (*n* = 1, 2, … ) for the time at the *n*th intersection of the disturbed trajectory with S after *I*(*t*) is added. The integration of (9) from 0 to *t*_*n*_ gives:

(10) $$\begin{array}{l} \int_{0}^{t_{n}}\left(\overset{∙}{\psi }-\frac{2\pi }{\tau }\right)dt=\int_{0}^{t_{n}}Z\left(\psi \right)I\left(t\right)dt \end{array}$$

The Poincaré section S generally mismatches with the isochron of ψ = 0, as shown in Figure 2, which induces the difference of ψ between the Poincaré section and isochron and thus $\int_{0}^{t_{n}}\overset{∙}{\psi }dt\neq 2n\pi$ (Imai and Aoyagi, 2016). However, because the disturbed trajectory approaches C as *t* → ∞, $\int_{0}^{t_{n}}\overset{∙}{\psi }dt=2n\pi$ approximately for sufficiently large *n*. This gives:

(11) $$\begin{array}{l} \int_{0}^{t_{n}}Z\left(\psi \right)I\left(t\right)dt=2\pi \frac{n\tau -t_{n}}{\tau } \end{array}$$

**Figure 3:**
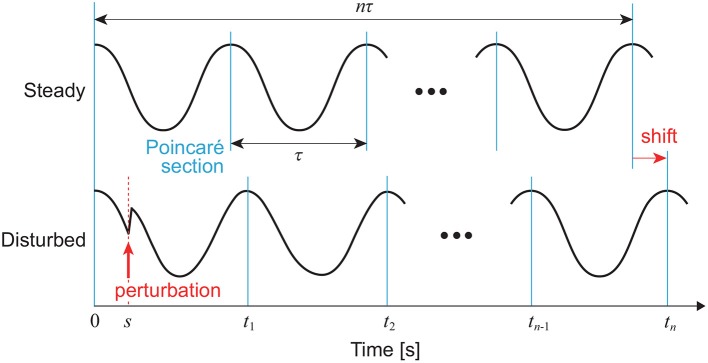
Phase shift by perturbation of a limit cycle oscillator at *t* = *s*. In this case, a positive peak condition is used for the Poincaré section.

The right-hand side can be obtained from the phase shift by the perturbation, as shown in Figure 3. For an impulsive perturbation at *t* = *s* (0 ≤ *s* < τ), which is given by *I*(*t*) = μδ(*t* − *s*) when μ is constant, (11) becomes:

(12) $$\begin{array}{l} \mu \int_{0}^{t_{n}}Z\left(\psi \right)\delta \left(t-s\right)dt=2\pi \frac{n\tau -t_{n}}{\tau } \end{array}$$

This gives,

(13) $$\begin{array}{l} Z\left(\psi \left(s\right)\right)=\frac{2\pi }{\mu }\frac{n\tau -t_{n}}{\tau } \end{array}$$

In this study, we calculated the PRC from (13) using our neuromusculoskeletal model, where we used the foot contact condition of the right leg (any of four contact points of the right foot is below the treadmill belt) for the Poincaré section S. In particular, our previous work (Funato et al., 2016) obtained the PRCs from human walking measurements by accelerating or decelerating the belt speed of a treadmill independently. To compare the simulation results with the human measurements, our model also walked on a treadmill and we obtained the PRCs for the acceleration and deceleration perturbations in the belt speed separately. More specifically, after the model achieved steady walking on a treadmill, we increased or decreased the belt speed by 0.1 m/s for 0.001 s once per trial ($\mu =2\pi \frac{\pm 0.1\cdot 0.001}{\nu \tau }$), where ν is the belt speed, and *n* = 50 so that the model achieved steady walking after being disturbed. We performed 100 trials by changing the perturbation phase to obtain the PRC. Furthermore, we used the models with and without phase resetting in the motor control model and compared the PRCs calculated from these models by referring to the PRCs obtained from measurements of human walking.

## 3. Results

**Figure 4:**
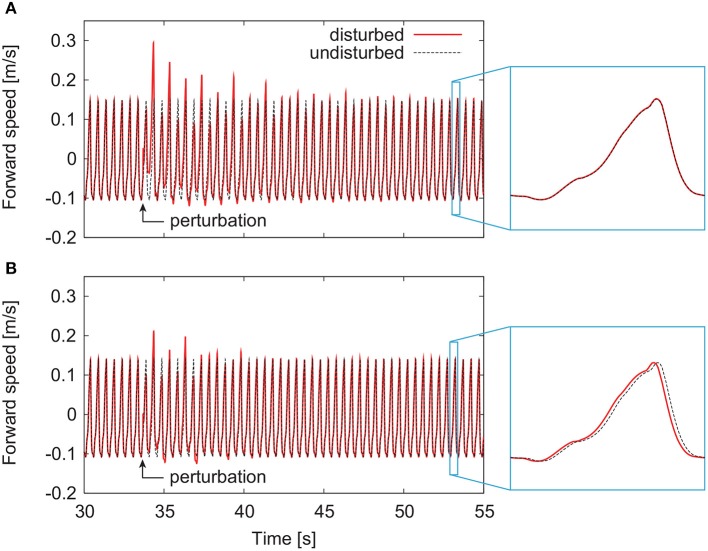
Responses of forward speed for the model **(A)** without phase resetting and the model **(B)** with phase resetting.

By modifying the motor control parameters from those in our previous work (Aoi et al., 2010), both the models with and without phase resetting achieved steady walking on a treadmill whose belt speed was 1.3 m/s. The locomotor behavior, especially the joint kinematics and muscle activities, were almost identical to those in our previous work (Aoi et al., 2010) except for the difference between walking over ground and on a treadmill. Figures 4A,B show representative responses of the forward speed for the models without and with phase resetting, respectively, after the models were disturbed. For both models, the forward speed fluctuated after the perturbation and then recovered to steady periodic behavior. Although the model without phase resetting had no shift of the locomotion phase after the recovery, the model with phase resetting had a phase shift.

**Figure 5:**
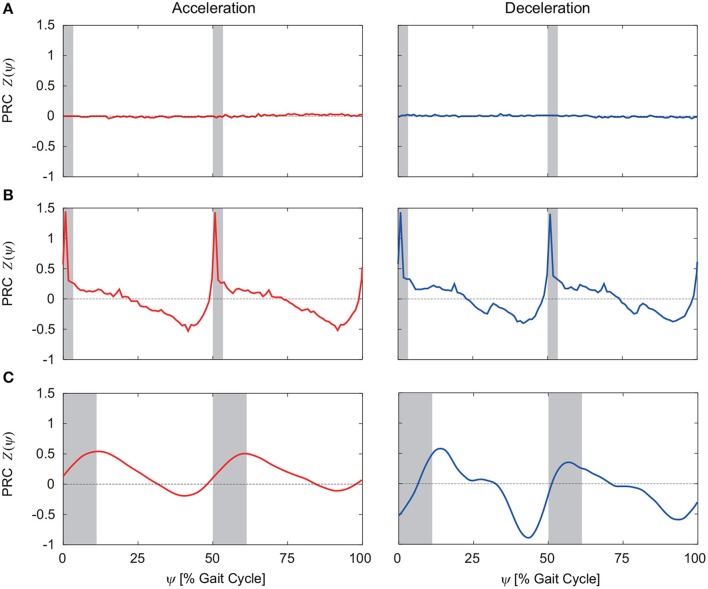
PRCs calculated for acceleration and deceleration perturbations for **(A)** the model without phase resetting, **(B)** the model with phase resetting, and **(C)** kinematic measurements of human walking. **(C)** is modified from Funato et al. (2016). 0 and 100% of the gait cycle represent right foot contact, and gray regions indicate the double-stance phase.

From 100 trials with different perturbation phases, we obtained the PRCs. Figures 5A–C show the PRCs calculated by acceleration and deceleration perturbations for the model without phase resetting, the model with phase resetting, and kinematic measurements of human walking, respectively. Although the PRCs for the model without phase resetting were zero irrespective of the perturbation phase for both types of perturbation, the PRCs for the model with phase resetting showed characteristic phase-dependent shapes. In particular, they intersected with the horizontal axis around the foot-contact timings and mid-stance phases. They had steep positive slopes around the foot contact and positive peaks in the double-stance phase. They also had gentle negative slopes after the double-stance phase and negative peaks before the next foot contact. Furthermore, the PRCs for the acceleration and deceleration perturbations were almost identical. These trends are similar to those in the PRCs obtained from the measurement of human walking.

**Figure 6:**
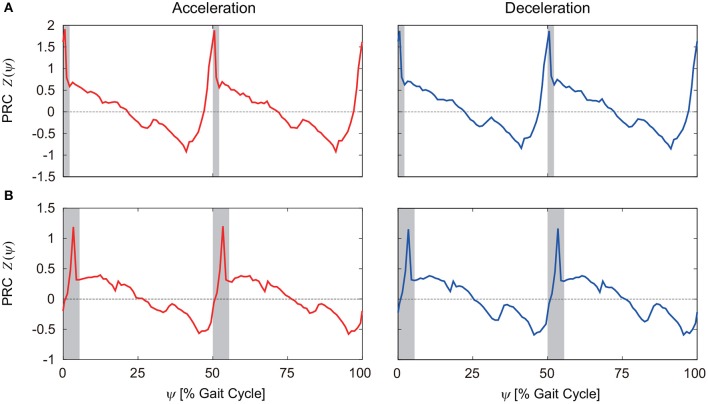
PRCs for acceleration and deceleration perturbations for the model with phase resetting when the steady belt speed was **(A)** increased and **(B)** decreased by 0.02 m/s. 0 and 100% of the gait cycle represent right foot contact, and gray regions indicate the double-stance phase.

To investigate the robustness of the obtained results, we examined how the PRC changes for different belt speeds and different motor control parameters, such as the gait frequency. Figures 6A,B show the PRCs for the model with phase resetting when the steady belt speed was increased and decreased by 0.02 m/s, respectively. Although there are some differences, the characteristic properties mentioned above remain unchanged.

## 4. Discussion

### 4.1. Contribution of Phase Resetting Based on Foot Contact

Our motor control model incorporated phase resetting induced by foot-contact information based on physiological evidence. In particular, cutaneous feedback was observed to contribute to phase shift and rhythm resetting behaviors in fictive locomotion of decerebrate cats (Duysens, 1977; Schomburg et al., 1998). Furthermore, spinal cats walking on a treadmill changed their gait, such as walking, trotting, and galloping, in accordance with the belt speed (Forssberg and Grillner, 1973; Orlovsky et al., 1999), which suggests that the tactile sensory information obtained by their feet from the belt influenced the locomotion phase and rhythm generated by the CPG (Duysens et al., 2000). In human walking, the timing of basic muscle activation patterns was also strictly linked to the foot-contact event (Ivanenko et al., 2006). Our model integrated the phase resetting mechanism with the muscle synergy hypothesis that suggested that a large portion of motor commands for walking is generated by the linear combination of five basic signals to solve the redundancy problem in motor control (Ivanenko et al., 2006). More specifically, phase resetting in our model just controlled the timing of the basic signals to determine the motor command using the foot-contact information, which is a very simple strategy. Despite the simple strategy, this timing regulation of the basic signals has been reported to produce various locomotor functions, such as the walk-run transition (Cappellini et al., 2006; Aoi et al., 2019), stepping over an obstacle (Ivanenko et al., 2005; Aoi et al., 2013), and split-belt treadmill walking (MacLellan et al., 2014; Fujiki et al., 2018). Hodgkin-Huxley style neuron model showed that the phase of the neurons' activity rapidly changed by external signals (Rybak et al., 2006a,b), which suggests that the neural system has a mechanism to quickly move the phase of neurons' activity. We would like to incorporate a more biologically detailed neuron model to further investigate the contribution of phase resetting in the future.

Electrical stimulation to the swing legs in cats (Forssberg et al., 1975; Forssberg, 1979) and humans (Belanger and Patla, 1984; Duysens et al., 1990) and mechanical stimulation in humans (Schillings et al., 1996, 2000) showed phase-dependent responses. In particular, stimulation early in the swing phase enhanced flexor muscle activities and extended the swing phase (elevating strategy), while stimulation late in the swing phase enhanced extensor muscle activities and advanced the foot-contact timing (lowering strategy) (Eng et al., 1994). From the intersection of the obtained PRCs with zero (Figure 5C), our previous work (Funato et al., 2016) showed that the mid-single stance phase extended in response to acceleration perturbations and the foot-contact timing advanced in response to deceleration perturbations, which correspond to the elevating and lowering strategies, respectively. In this study, we incorporated the phase resetting mechanism that modulates the timing of the motor command based on the foot-contact information. This mechanism is related to the lowering strategy. Despite not incorporating the elevating strategy, our model had a PRC shape similar to that for humans not only for deceleration perturbations but also for acceleration perturbations (Figures 5B,C). That is, application of only one of these two strategies allowed the model to reproduce the PRCs for acceleration and deceleration perturbations in humans. In the future, we would like to incorporate the elevating strategy in our motor control model to further clarify the adaptive rhythm control mechanism in human walking.

### 4.2. Calculation of PRC

To calculate the PRC from kinematic measurements, mainly two methods have been proposed. One is the impulse method that uses single-impulse perturbation, and the other is the weighted spike-triggered average (WSTA) method, which uses sequential pulse perturbation with zero mean and no temporal correlation (Ota et al., 2009). Our previous work (Funato et al., 2016) used both of these methods to calculate the PRCs for human walking. Because the impulse method required many trials that exhausted the subjects, the obtained PRCs had large deviations and low temporal resolution and could not show characteristic properties. The WSTA method improved the PRCs, and clear phase-dependent shapes could be resolved (Figure 5C). However, it still has limitations with regard to obtaining precise PRCs. For example, two positive peak timings of the PRC for the deceleration perturbation differed. In addition, the acceleration and deceleration perturbations showed some differences in the PRC, and it was difficult to determine whether they were actually different or due to limitations of the method. In particular, although the PRC was analytically derived under the assumption that the perturbation is sufficiently small, the perturbation must be large to reduce the influence of measurement noise in human experiments. In this study, we used a mathematical model to obtain the PRCs for human walking. Because of the high reproducibility of the simulation results, we obtained accurate PRCs for the model using arbitrarily small and short perturbations by the impulse method. Our model showed identical PRCs for the acceleration and deceleration perturbations. The modeling approach using the PRC has an advantage for improving our understanding of the underlying rhythm control mechanism.

### 4.3. Limitations of Our Model and Future Work

The PRC for the model with phase resetting had a similar shape to that of the PRC for humans, and it supports the hypothesis that phase resetting contributes to adaptive rhythm control in human walking in comparison with the PRC for the model without phase resetting. However, our model has limitations for accurately reproducing the PRC for human walking. For example, the PRC for the model with phase resetting had much steeper positive peaks in the double-stance phases compared to the PRC for humans (Figures 5B,C). This is possibly because four discrete points on each sole were used for the foot-contact model. Due to the discrete points, perturbations in double-stance phases induced sudden changes in locomotor behavior and caused the steep positive peaks in the PRC. In addition, our model showed short double-stance phases compared to actual human walking (Figures 5B,C), which is mainly due to no phalangeal joint in our foot model. However, the PRCs for the model and humans had similar characteristics in the double-stance phase, such as steep positive slopes around the foot contact and positive peaks located in the double-stance phase.

Although we incorporated the phase resetting mechanism in the motor control model, other sensory-motor coordination mechanisms also play a role in human walking. For example, although we focused on the swing-to-stance phase transition using the foot-contact information, the stance-to-swing phase transition has been suggested to include important sensory-motor coordination mechanisms (Ekeberg and Pearson, 2005; Pearson et al., 2006; Dzeladini et al., 2014; Song and Geyer, 2015), such as the unloading rule that uses force-sensitive afferents in the ankle extensor muscles (Duysens and Pearson, 1980; Whelan et al., 1995) and the hip extension rule that uses position-sensitive afferents from the hip (Grillner and Rossignol, 1978; Hiebert et al., 1996). In addition, although this study changed the belt speed of a treadmill to disturb human locomotor behavior, other types of perturbations, such as pulling on the swing leg, have been used (Kobayashi et al., 2000; Nessler et al., 2016). Because the PRC depends on the perturbation, we would like to incorporate other sensory-motor coordination mechanisms and perturbations to further clarify adaptive rhythm control in human walking.

## Data Availability Statement

The datasets generated for this study are available on request to the corresponding author.

## Author Contributions

SA developed the study design. DT performed computer simulations and analyzed the data in consultation with SA, TF, SF, KS, and KT. DT and SA wrote the manuscript and all the authors reviewed and approved it.

### Conflict of Interest

The authors declare that the research was conducted in the absence of any commercial or financial relationships that could be construed as a potential conflict of interest. The handling Editor declared a shared affiliation, though no other collaboration, with some of the authors DT, SA, KS, and KT.
